# Insights into the Skeletonization, Lifestyle, and Affinity of the Unusual Ediacaran Fossil *Corumbella*

**DOI:** 10.1371/journal.pone.0114219

**Published:** 2015-03-30

**Authors:** Mírian L. A. Forancelli Pacheco, Douglas Galante, Fabio Rodrigues, Juliana de M. Leme, Pidassa Bidola, Whitey Hagadorn, Marco Stockmar, Julia Herzen, Isaac D. Rudnitzki, Franz Pfeiffer, Antonio C. Marques

**Affiliations:** 1 Department of Biology, Federal University of São Carlos, Sorocaba, Sao Paulo, Brazil; 2 Brazilian Synchrotron Light Laboratory, Campinas, São Paulo, Brazil; 3 Institute of Geosciences, University of São Paulo, São Paulo, Brazil; 4 Department of Physics and Institute for Medical Engineering, Technische Universität München, Garching, Germany; 5 Department of Earth Sciences, Denver Museum of Nature & Science, Denver, United States of America; 6 Institute of Materials Research, Helmholtz-Zentrum Geesthacht, Geesthacht, Germany; 7 Institute of Geosciences, Federal University of Pará, Belém, Pará, Brazil; 8 Institute of Biosciences and Center for Marine Biology, University of São Paulo, São Paulo, Brazil; Laboratoire Arago, FRANCE

## Abstract

The Ediacaran fossil *Corumbella* is important because it is hypothesized to be a scyphozoan cnidarian, and thus might be one of the rare examples of bona fide Neoproterozoic animals. Unfortunately, its mode of life, style of skeletonization, and taxonomic affinity have been very controversial. Here, we use X-ray micro-CT, SEM, and taphonomic analysis to compare preservational modes of *Corumbella*, in order to better understand the symmetry, mode of construction, preservational style, and taxonomy of this group. Results suggest that articulated and disarticulated specimens of *Corumbella* from the Ediacaran of Brazil, Paraguay, and the United States, although sometimes preserved very differently, represent the same taxon—*Corumbella werneri*. Corumbellids had a thick but flexible theca and probably lived with their basalmost part anchored in the sediment, much like *Conotubus*. When considered together, these results suggest that *Corumbella* was one of the first animals to build a skeleton, employing a lamellar microfabric similar to conulariids.

## Introduction

**Fig 1:**
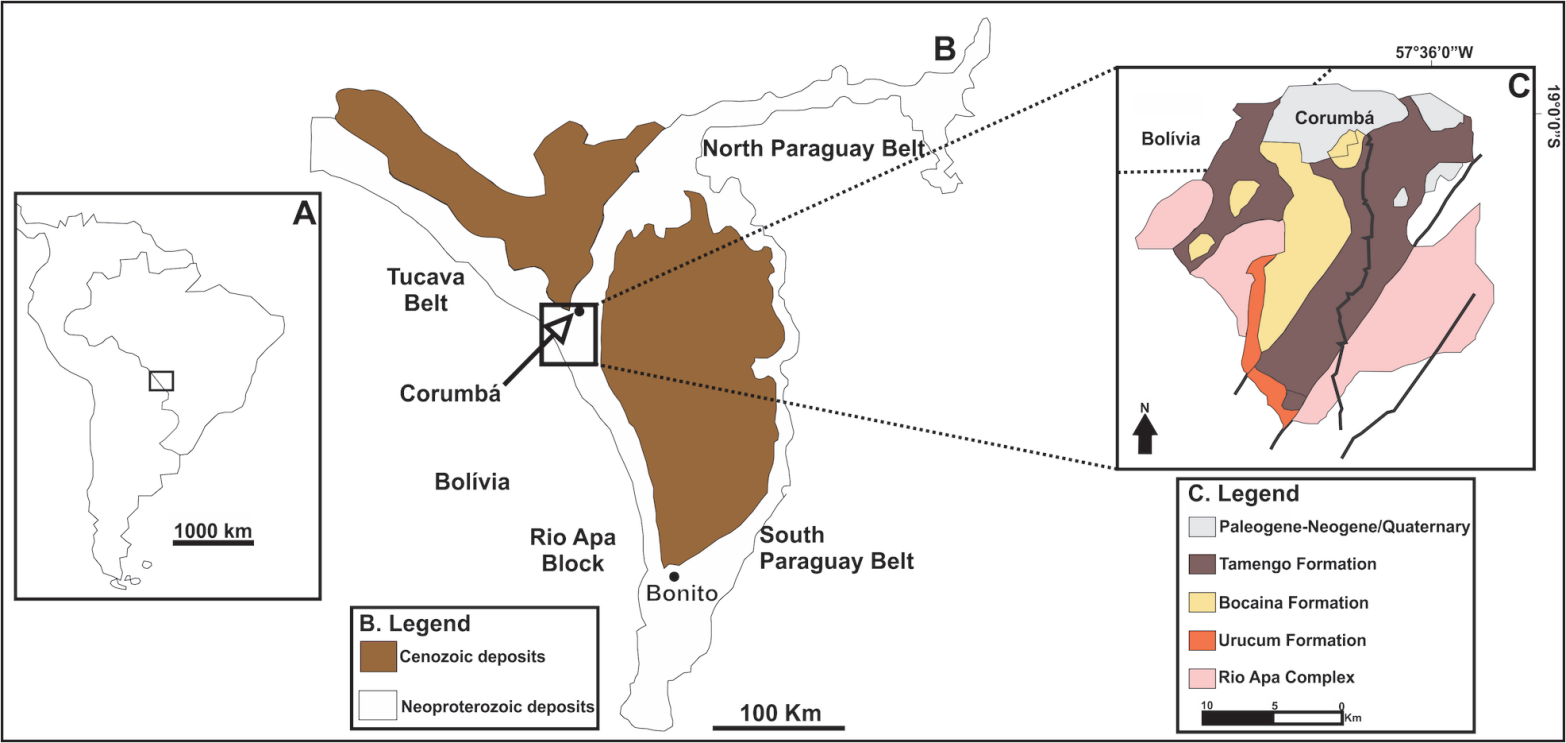
Simplified map of South America (A) with detail of the geological map of the Paraguay Belt (B), and Corumbá Group (C) Modified from Oliveira, 2010, [48].

The Corumbá Group of Mato Grosso do Sul, southwest Brazil (Fig. 1), has the most diverse assemblage of Neoproterozoic fossils in South America [1, 2]. These include vase-shaped microfossils, algae (*Tyrasotaenia* sp.), and metazoans (*Corumbella werneri*, *Cloudina lucianoi*) [3–8].

**Fig 2:**
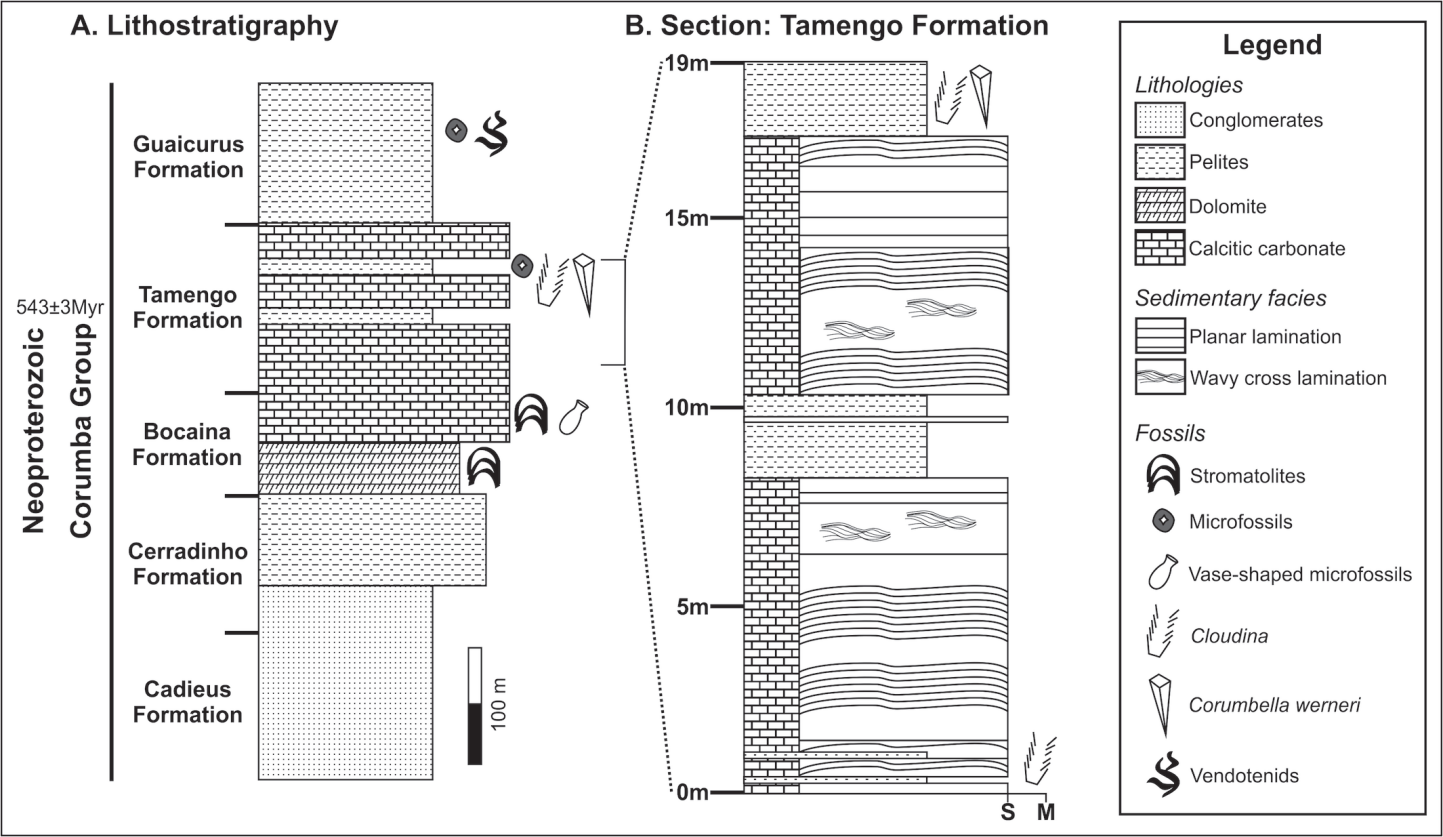
Lithostratigraphic column for Corumbá Group and section for the upper part of Tamengo Formation (Neoproterozoic) at the Saladeiro quarry. Detail of the position where *Corumbella werneri* and other fossils occur. M—mud; S—sand. Modified from Warren *et al*., 2012, [17]; and Morais, 2013, [49].

Perhaps the most enigmatic of these taxa is *Corumbella werneri*, a polyhedral multi-segmented flexible organism. It has been associated with scyphozoan cnidarians or other extinct cnidarian clades, such as the conulariids [4–6, 9]. This fossil is abundant in the limestone mines located in Corumbá and Ladário, Brazil, where it occurs in the Ediacaran Tamengo Formation (Fig. 2). Co-occurrence of *C*. *werneri* with the Ediacaran index fossil *Cloudina* suggests that the Tamengo Formation fossils are part of the Ediacara biota [2, 8, 10–12].

Although the Tamengo Formation lacks other biomineralized Ediacaran fossils, such as *Namacalathus* [13, 14] and *Namapoikia* [15], its shales and marls show a range of preservational style of fossils. *Corumbella* also occurs in the grainstones and shales of the coeval Itapucumi Group of Paraguay [16, 17], as well as in the sandstones of the Wood Canyon Formation of the USA [9]. In these three deposits, *Corumbella* occurs as casts, molds, and body-fossils, in bed-parallel or bed-perpendicular orientations, and as single specimens or as clasts within pavements.

This diversity of preservational styles, together with an abundance of new corumbellid specimens, allows reassessment of the ecology, biology, and taxonomy of this organism, and places it among the novel evolutionary changes that occurred during the end of the Ediacaran period, such as the rise of skeletonized animals and carnivores predators [15, 17]. New specimens of *Corumbella* were collected from the Corumbá and Ladário exposures in Brazil, which were analyzed and compared to previously described specimens from Brazil, Paraguay and the USA, aiming to better understand its taxonomy and paleobiology.

## Historical Taxonomy and Morphology

**Fig 3:**
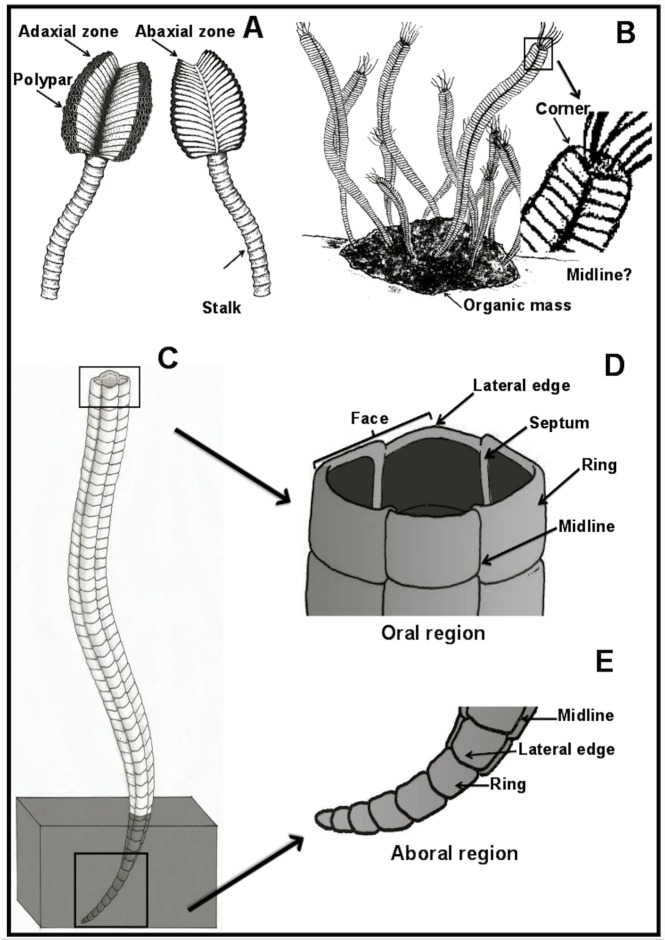
Models for Corumbella werneri. (A) Reconstruction of *Corumbella werneri* proposed by Hahn *et al*. (1982) [4], showing a stalk and polypars (modified by Wilson Soares Jr). (B) Reconstruction of *Corumbella werneri* as a colonial sessile organism, as interpreted by Babcock *et al*. (2005) [5] (modified by Abner Santos). Detail of the square symmetry. (C) Reconstruction of *Corumbella werneri* proposed by Pacheco *et al*. (2011) [6] and improved in this work (draw by Abner Santos). (D) Detail of the oral region, lateral edges, faces, septa and midline. (E) Aboral region with unisseriated rings on the attachment portion.

The first taxonomic description and paleoecologic and paleobiogeographic interpretations of *Corumbella werneri* were based on material from marls and shales of the Saladeiro quarry (Fig. 2), located in Ladário, adjacent to Corumbá. Originally, this material was assigned to a new subclass Corumbellata, and placed among the Cnidaria Scyphozoa [4]. Two distinct body regions were recognized in *Corumbella werneri* (Fig. 3A). The first is the proximal region, consisting of a curved, elongate, unbranched tubular periderm (or stalk), designated as the “primary polypar”, which is made up of isolated, and hypothesized, chitinous rings, externally and internally reinforced on the sides with four small, short internal sclerosepta. The second is a distal region, consisting of a biseriate arrangement of secondary, contiguous polypars, each formed by a small, distinct chitinous periderm tube, with no clear ring formation or visible sclerosepta. Secondary polypars are merged to each other at the adaxial surface. The set of two secondary polypars has a morphology similar to the primary polypar, differing only in the diameter. The biseriate portion is slightly wider and shorter (8–10 cm) than the uniseriate one. An apical part (here named attachment portion) in the primary polypar was not recognized (though basalmost oriented).

In the first description [4], it was also highlighted the internal four-fold radial symmetry of *C*. *werneri*, which was implied by the arrangement of sclerosepta in cross-sections of the primary polypar. This character was considered to be similar to the internal arrangement found in the polyps of the recent scyphozoan genus *Stephanoscyphistoma* Jarms, 1990 (Coronatae; former *Stephanoscyphus* Allman, 1874), which was used to classify *Corumbella* as a Scyphozoa (phylum Cnidaria). However, they considered the transition from the proximal uniseriate primary polypar to the distal biseriate secondary polypars of *Corumbella werneri* to be a distinct character and, on the basis of this hypothesis, the authors erected the family Corumbellidae, order Corumbellida, subclass Corumbellata [4, 10].

**Fig 4:**
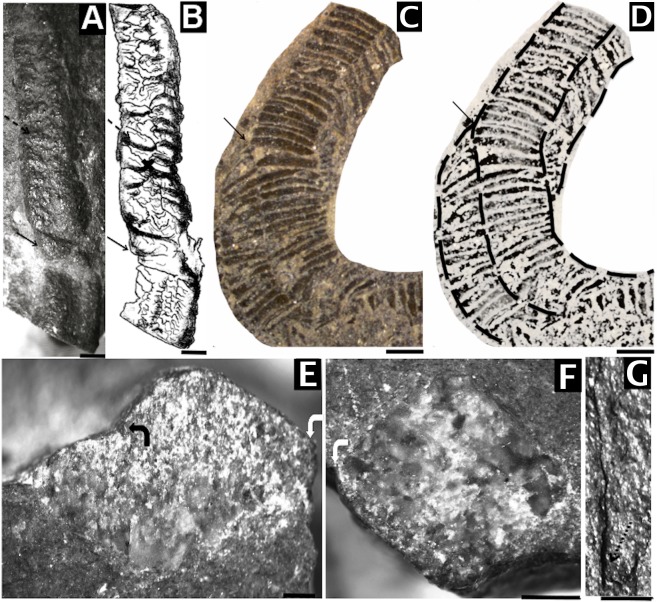
Corumbella werneri, Wood Canyon (USA) and Tamengo Formation (Brazil). Morphology. (A) LACMNH 12802 (Wood Canyon Formation). Polyhedral specimen, covered with desert varnish. Observe rings (black dashed arrow) alternately converging on the midline (marked with an “X” in the draw), in continuity with the lateral edges. Detail of the helical twist (black arrow). (B) Draw representing (A) with detail of the rings (black dashed arrow); midline (marked with an “X” in the encounter of two rings in the face) and torsion (black arrow). (C) and (D) GP1E-5808b (Tamengo Formation). Note torsion in the two-dimensional tube (black arrow) (C) and detail of the helical twist represented in (D) (black dashed lines). (E) LACMIP loc. 17130 (Wood Canyon Formation). End section quadrangular pressed torsional in (A). Observe lateral edges (white curved arrow) and septa (black curved arrow). (F) LACMNH 12802. Quadrangular section untwisted end in (A). See lateral edge (white curved arrow) (G) LACMNH loc. 17130. Aboral region. Detail for lateral continuity of the rings (black dotted arrow). Scale: 1 mm.

It was described [9] from the Ediacaran portion of the lower member of the Wood Canyon Formation (USA), but not named, “*Corumbella* n. sp.”, remarking that the two specimens they had found had a single longitudinal groove along the midline and were spirally twisted (Fig. 4A, B). Authors motivate the comparisons between *C*. *werneri* and USA specimens [9] by the original description [4], now considered outdated by the more recent studies [5, 6]. They do not consider *Corumbella* n. sp. conspecific with *C*. *werneri*, which in original description [4] lacks a helical twist and has transverse ornamentation, secondary polypars and circular geometry in cross sections (Fig. 4 E, F) [4]. Therefore these specimens were not assigned to *Corumbella werneri* (Brazil). *Corumbella* n. sp. was assigned to the Cnidaria [9] because the specimens were similar in symmetry and external ornamentation with conulariids, a group previously allied with Scyphozoa, Cnidaria [18–26].

Then, a new morphological and taxonomic interpretation for *C*. *werneri* [5] was presented, in which a tetramerous elongated narrow tube would be secreted from an “apical attachment region” (referred as the apex, though basalmost oriented) (Fig. 3B). The animals were hypothesized to be benthic colonial predators fixed to the substrate by an “organic mass”. The reinterpretation of *C*. *werneri* as tubular and its budding reproduction strengthen their similarity with the modern *Stephanoscyphistoma*, and possibly with the extinct conulariids [5]. However, key morphological features essential for testing this paleoecological and taxonomic interpretation were described but not indicated in the described material [5], including midline thickening, attachment structures, oral region, the “organic mass”, and rectangular sections showing four-fold symmetry.

Other researchers [16] described *Corumbella werneri* from Itapucumi Group, Paraguay, as long and flattened fragments made up of articulated, apparently organic, narrow, annular elements, possibly corresponding to transverse rod-like skeletal elements that occur in conulariid scyphozoan cnidarians.

Recently, geometric modelling and taphonomic analysis were used to refine the understanding of the original specimens of *Corumbella werneri* and to carry out a more detailed taxonomic analysis of the group [6]. It was hypothesized that *Corumbella* would have a uniseriate aboral region that graded to a polyhedral geometry bearing lateral edges (Fig. 3C-E). The theca was constructed of polygonal rings merged into each other at a midline, the central region of the faces, as apothem of a pyramid, the side height of a lateral face—see ref. [6], (Fig. 7, pg. 278); (Fig. 3D).

However, there are still aspects of the morphology of *C*. *werneri* that are unknown, such as the nature of inner structures, its aboral region and skeletal micro and ultrastructure. For example, important questions are: Did corumbellids have septa? What was the microfabric or mineralogy of their theca? And did they lay on or attach to the seafloor? In the past, it was difficult to answer these questions because the fossil’s attachment region or internal features are usually obscured by sediment or flattening during compaction, or because specimens previously studied were not well enough preserved to show primary microstructural details. Using microfocus X-ray computed tomography (microCT) in tandem with SEM and ultrastructural analysis of newly collected material, it is possible to fill some of these knowledge gaps and gain insights into the paleobiology, paleoecology, and phylogenetic affinity of these fossils.

## Geological Background

The Corumbá Basin outcrops in the southern part of the Paraguay Belt, Brazil (Fig. 1). In this region, the base of the sequence consists of the Cadiueus and Cerradinho formations, which were deposited in an initial continental rift basin, before the widespread of carbonate deposition [2]. Overlying these strata are the Bocaina, Tamengo and Guaicurus formations, which were deposited in a stable marginal basin [27–29]. The Bocaina Formation comprises stromatolitic dolostones and subordinate phosphorites that grade to black limestones, marls and shales of the Tamengo Formation. The Guaicurus Formation, which is the youngest unit of the Corumbá Group, is comprised of thick siltstones and shales overlying the Tamengo Formation (Fig. 2) [28, 32].

The Bocaina, Tamengo, and Guaicurus formations represent the evolution to open shelf sedimentation in a post-rift to drift stage, as indicated by the occurrence of phosphorite at the top of the Bocaina Formation and the presence of the fossil *Cloudina* in the Tamengo Formation [2, 28], similar to other late marine Ediacaran environments, such as Namibia and Canada [13, 14, 32].

The *Corumbella*-bearing portion of the Tamengo Formation was dated about 543±3 Ma [30, 31] by U-Pb SHRIMP zircon form an ash bed interbedded in the upper Tamengo Formation (Fig. 2B). This age collaborates the correlation with records of Precambrian-Cambrian boundary in previous age constrains from Siberia and Namibia around 543Ma [33]. Also in Ara Group of Oman, the anomaly negative carbon excursion and abrupt last appearance of *Cloudina* and *Namacalanthus*, are dated by U-Pb zircon age in ash interbedded, directly define these event to be at 542±3Ma [34]. This parameters allow confirm that the *Corumbella* record and the age in Tamengo Formation agree very well with Precambrian-Cambrian boundary showed in previous work in distinct records.

The widespread carbonate deposition of the Tamengo Formation is related to a transgression event also observed in the calcareous grainstones of the Tagatiya Guazu Formation (Itapucumi Group, Paraguay). This unit is thought to be a wave- and tide-dominated depositional environment that represents a shallow tidally-influenced setting on a rimmed carbonate ramp [16], where the association of *Cloudina* fossils with thrombolites together with remains of *Corumbella* [17] were found.

The presence of the *Cloudina* and *Corumbella* in the Itapucumi Group and Tamengo Formation confirms that these two units are correlated [16]. In Corumbá, *Cloudina* occurred in a shallow, protected carbonate setting [16], whereas *Corumbella* lived in a calm terrigenous environment, indicated by its abundance in marls. In Paraguay, these animals probably co-occurred in environments with similar conditions of water temperature, depth, and salinity [17]. The siltstones and very fine-grained sandstones of the lower member of the Wood Canyon Formation in Nevada, USA, also bear *Corumbella* and *Cloudina*. Based on chemostratigraphic and biostratigraphic correlation with other radiometrically dated units, it was proposed that the fossil-bearing strata of the Wood Canyon Formation were probably deposited in the latest Ediacaran time, in shallow subtidal nearshore marine environments [9].

## Material and Methods

### Examined collections

419 samples were analyzed: 401 from Brazil, deposited at the Scientific Collection of the Geoscience Institute of the University of Sao Paulo (IGc/USP) (“GP/1E”), 2 from the Collection of the Laboratory of Paleobiology (IGc/USP), 12 from the Collection of the Department of Paleontology of the National Department of Mineral Production (DNPM), Rio de Janeiro (“DGM”), and 2 from the United States, deposited at the Los Angeles County Natural History Museum (“LACMNH” and “LACMIP”). Regarding the specimens from Paraguay, published data were used [9].

### X-ray Microtomography

X-ray imaging can help the visualization of inner structures of fossils because X-rays penetrate opaque minerals and matrices without destructive sectioning of specimens [35]. The contrast between two features of X-ray imaging is based on differences in the attenuation of X-rays, as they pass through materials of different densities and elemental compositions. Computed tomography allows the use of multiple projected images recorded while the sample is rotated around an axis to assess the three-dimensional geometry and internal structures of fossils.

Specimens from the Brazilian and USA collections were examined using a “V|tome|x” (Phoenix X-Ray/General Electric) micro-CT scanner, at 90 kV and 130 μA. Tomographic reconstructions were based on 2000 single projection images equally spaced through 360°. Projection images were captured using a 2000 x 990 pixel coupled scintillator-CCD with 2 s exposure time. The resulting effective voxel size due to geometrical magnification was 12.3 μm^3^.

### Scanning electron microscopy and Energy Dispersive Spectroscopy (SEM/EDS)

The microstructural analyses of fossils were also performed by the means of SEM/EDS at Brazilian Nanotechnology National Laboratory (LNNano). Fossils were examined using a SEM FEI Quanta 650 FE in secondary electrons detection mode, with acceleration voltages of 10 and 20 kV. EDS was performed using an X-Max detector in mapping mode, in order to detect the distribution of calcium in exoskeleton and rock matrix.

### Morphological description and systematic analysis of specimens

Morphological and microstructural features are described using the published terminology [4–6, 9, 12, 20, 22]. Whenever possible, we followed the recommendations of other authors [36, 37], *viz*. (a) the taxonomic study of invertebrate fossils is based on the highest number of samples available, aiming to collect a broad spectrum of morphological and taphonomic data; (b) the study prioritizes the examination of specimens with different types of preservation; (c) comparison is based on morphometric characters of specimens with similar preservation.

## New Data on Structure and Morphology

**Fig 5:**
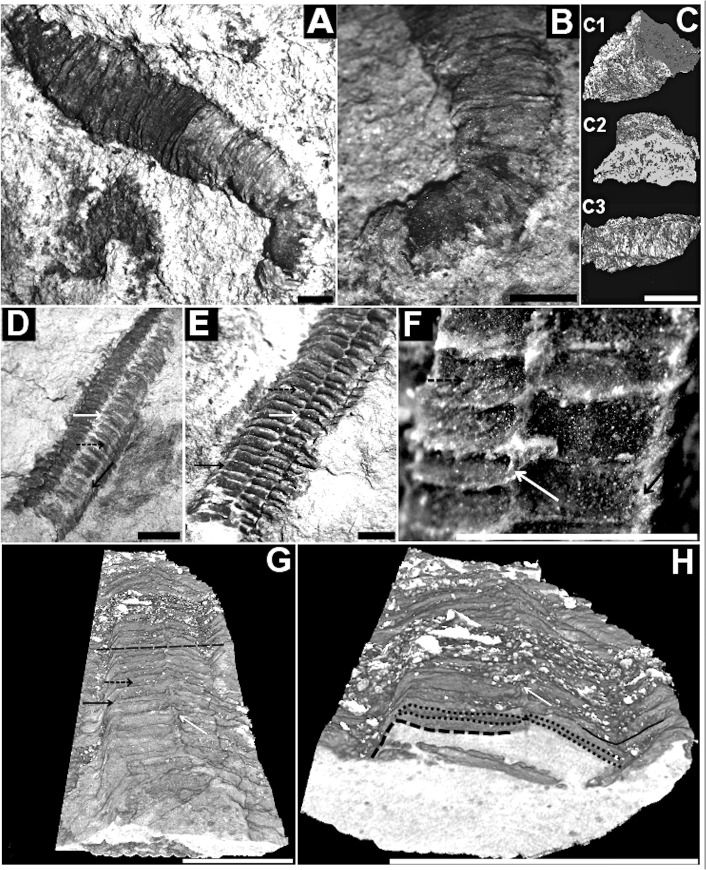
Corumbella werneri: morphology and modes of life. (A) GP1E-4216: *Corumbella werneri* and (B) attachment region evidenced by (C) 3D-rendered microCT. (C1) shows a transverse-lateral section of (B). In (C1), it is possible to observe the conical morphology of the final attachment region, obliterated by the rock matrix in (B) (black arrow). (C2) is a detail of the attachment region in transversal section. (C3) is a lateral view of the final attachment region. (D) GP1E-4109: external mold of (E), GP1E-4210: internal mold with prismatic geometry and almost square in cross section. Note lateral edge (black arrow), midline (white arrow) and the alternate disposition of rings (black dashed arrow) across midline, on the face. (F) Zoom of specimen (E) to observe the “u” alternate disposition of rings across midline (white arrow), rings (black dashed arrow) and the continuity of rings on the lateral edges (black arrow). (G) and (H) MicroCT of (E). (G) Transversal section (black dashed line) showing a folded polyhedral tube in (H). Details of the rings (black dotted line), lateral edges (black dashed line) and open folded lateral edge (black line). Scale: 1mm. (A), (B) and (E): reprinted from Pacheco *et al*. (2011) [6] under a CC BY license, with permission from Luis Alcalá, original copyright 2011.

**Fig 6:**
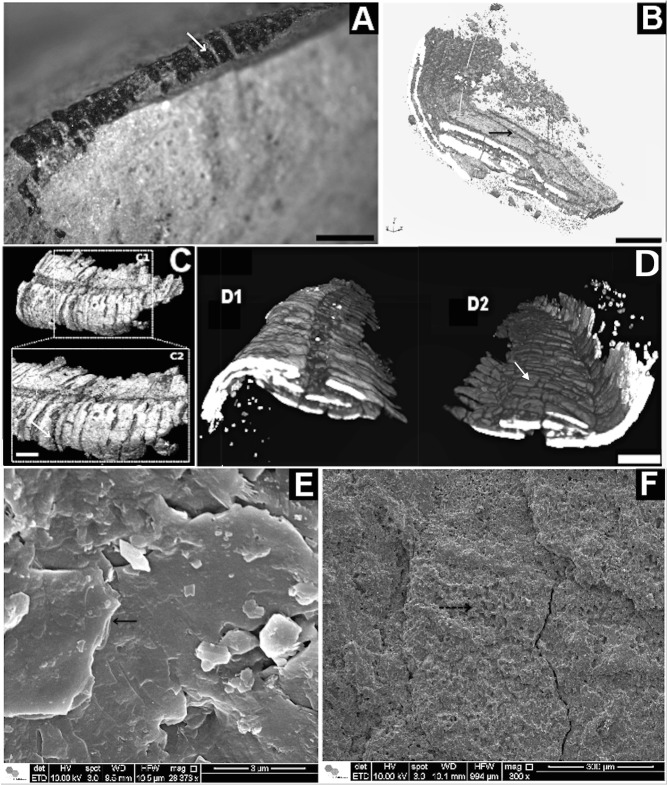
Corumbella werneri: ultrastructure of theca. (A) GP1E-574a: three-dimensional specimen with theca. (B) 3D-rendered microCT of *Corumbella* theca,flipped by 180° compared to (A): interior view and detail for lamellae microfabric and plates (black arrow). (C) 3D-rendered microCT of *Corumbella* theca (A) without flipping in C1 and C2, with details of rings (white arrow). (D) compression and fragmentation along theca. D1 shows a transversal section in the fossil structure. Flipped by 180° of it produces D2, with details of small breakages (white arrow). (E) Details of lamellar plates by SEM (black arrow) and (F) pores in plates (black dashed arrow). Scale: 1mm.

**Fig 7:**
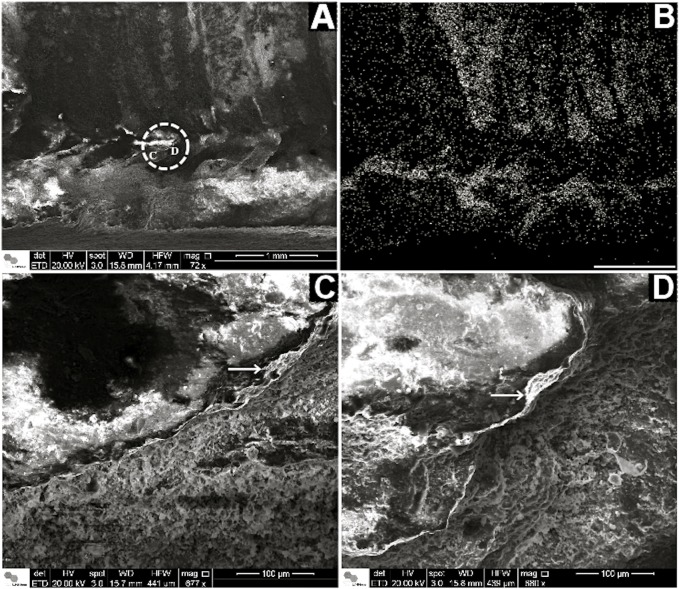
Corumbella werneri: ultrastructure of theca. (A) SEM of a longitudinal section of a *Corumbella* theca. Dashed circle marks a detail of a sectioned ring. (B) EDS mapping of (A). Here it is possible to observe higher concentration o calcium in fragments of theca (represented by white dots) in comparison to the rock matrix and molds of fossil without fragments. (C) and (D) are details of the white dashed circle in (A), showing micro layers in *Corumbella* theca (white arrow). Scale for B: 1mm.

The first reconstructions of *C*. *werneri* [4] were based on some specimens that were three-dimensionally preserved. The type-specimens, as well as types, paratypes, and comparable specimens from Paraguay and the USA, were carefully re-examined. In this regard, microCT images and some additional data on SEM/EDS proved to be essential in showing some new aspects of the morphology and taphonomical implications, such as deformation of morphologic features, that could result in wrong taxonomic descriptions. In this sense, it was possible to observe in *C*. *werneri* specimens: the morphology and arrangement of the aboral portion or anchor in the substrate, aspects of geometry and construction (Fig. 5), internal exoskeletal, mineralogy, lamellar microfabric, compression and fragmentation (Fig. 6, 7).

Structural and morphological attributes were considered here in order to compare specimens from South and North America.

### Morphology

**Fig 8:**
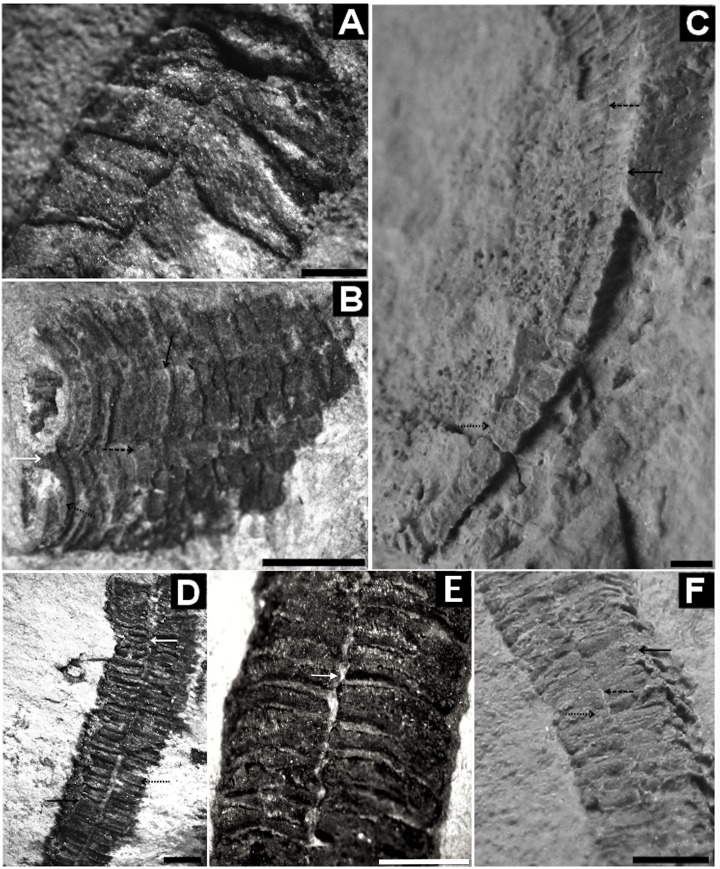
Corumbella werneri: morphology and modes of life. (A) GP1E-4089: oral region. (B) GP1E-4077: Inflated tube. Detail of a face with septum (white arrow), midline (black dashed arrow), rings (black dotted arrow) and lateral edge (black arrow) relatively compressed. (C) DGM-5601-I: specimen used in the original description by Hahn *et al*. (1982). Internal mold, recurved. It is observed the aboral region, uniseriate, without evidente midline in the (incomplete) attachment region. These rings (black dotted arrow) grade to an approximately polyhedral portion, with midline (on the face) (black dashed arrow) and lateral edge (black arrow). Notice a break in the longitudinal mid-distal portion of the fossil. (D) GP1E-4204: tube with longitudinal breakage, where it is evident the septum (white arrow), lateral edge (black arrow) and rings (black dotted arrow). (E) GP1E-3093: internal portion of folded tube, thick exoskeleton, where it is possible to see the septum (white arrow) formed by the inner part of the alternate rings (black dashed arrow). (F) DGM-5606-I: internal mold, three-dimensional, with midline (black dashed arrow), rings (black dotted arrow) and fragments of theca on the lateral edge (black arrow). Scale: 1mm. (A), (B), (C) and (D): reprinted from Pacheco *et al*. (2011) [6] under a CC BY license, with permission from Luis Alcalá, original copyright 2011.

The bipartite region of *C*. *werneri* body (Fig. 3A) may have originally been attributed to fragments of three-dimensional or two-dimensional uniseriate tubes. These tubes were interpreted as part of a primary polyp or stalk (DGM-5601-I, Fig. 8C), and the biseriate region was interpreted as polypars (DGM-5601-I, 5606-DGM-I, Fig. 8C, F). In previously analyzed samples, these parts were often arranged separately. In this paper, they are interpreted as fragments, sometimes from the same individual. Based on this study, it appears that *Corumbella werneri* is a polyhedral tube (Fig. 5D, E) with oral and aboral regions, and that the aboral region has a conical attachment (Figs. 3C-E and 5A-C) see ref. [6], pg. 277.

Three-dimensional modeling of *Corumbella* specimens from the Wood Canyon Formation indicates that their structure and anatomy are strikingly similar to the Tamengo Formation specimens. They have an elongated tubular polyhedral (Fig. 4A) appearance and are formed by four faces and four lateral edges, showing an almost quadrangular cross-section (Fig. 4F). The rings are inconspicuous, but it is possible to see they are polygonal, and merge with each other across midline (Fig. 4A, B). There are lateral edges (Fig. 4E, F) and faces with no clear demarcation of midlines, but with internal septa (Fig. 4E). These specimens were stretched, and fossilized in sandstone, and the fragments of the theca are partly obscured by desert varnish. Unlike the Tamengo Formation’s specimens, the Wood Canyon Formation’s specimens have a thinner theca without longitudinal striations, and there is no evidence of the oral region in these specimens. One specimen has an aboral region (Fig. 4G), but not a clear attachment portion.

### Structure and ultrastructure

**Fig 9:**
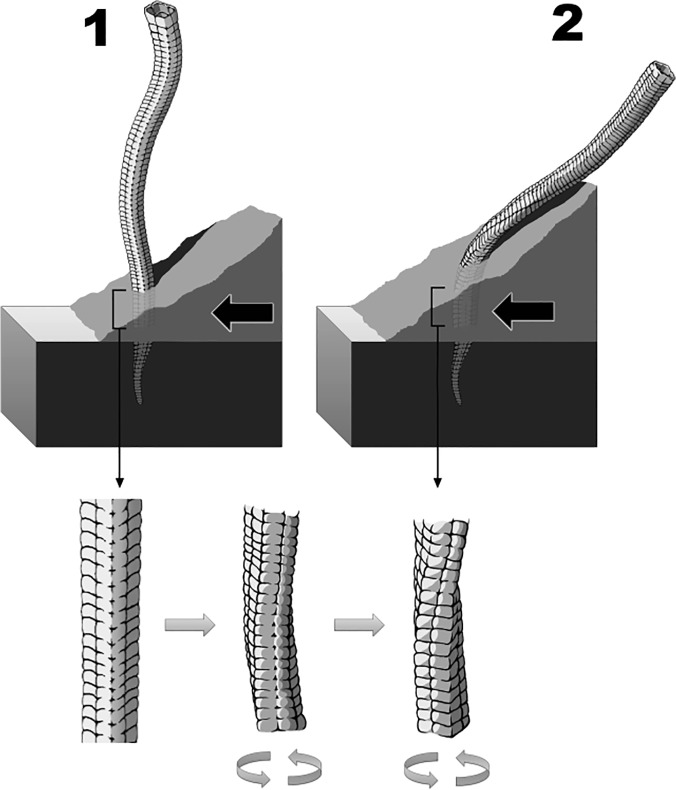
Simplified model for the mechanical origin of the torsion observed on the Corumbella specimen from USA (LACMNH 12802), which is dependent of the physical conditions of the animal and/or of the external forces. Subfigures 2 to 1 correspond to different stages: black arrows show a reconstitution from helical twisting happening when a *Corumbella* tube becomes partially buried (stage 2) until a possibility of *Corumbella* life mode (stage 1). Gray arrows point details of twisting until stage 2. Gray circular arrows show possibilities of twisting degrees in tubes.

Unlike completely biomineralized integuments of other metazoans, biomineralized Ediacara fossils often have weak or unusually flexible integuments (*e.g*. *Cloudina*). Thecae of *C*. *werneri* are similar—they show twisting and stretching in response to deformation, which suggests they had some plasticity. For example, both specimens from the USA and Brazil show torsions along the tube (Fig. 4A-D). In Brazilian specimens, the basalmost part ends directly in the substrate (Fig. 5A-C), just as described for *Conotubus* [38], suggesting that adults might be benthic. *Corumbella werneri* from the USA also has an aboral region, recognized as a basalmost ending attachment portion. In this sense, the presence of helical twisted regions suggests that these torsions result from mechanical processes acting on the pyramidal structure of the tube. When twisting a polyhedron, this usually happens due to its asymmetric geometry and/or due to mechanic *torques* (Fig. 9). In the case of *Corumbella*, helical twisting could have happened if the animal was partially buried when it was subjected to an external twisting force (such as a current or turbid flow that might also bury the organism). Hence, the response of tube to external forces depends not only on the rigidity of the theca, but also on their internal filling. Wood Canyon specimens are commonly filed with sediments coarser than the surrounding matrix, which causes a differential mechanical response to external *torques*, such as those produced on the burial process. Torsions can result from this transfer of forces from the matrix sediments to the structure, which can be more pronounced depending on the grain size of the sediment.

Since the structure of the tube was not entirely rigid, but fairly flexible in some twisted specimens, only a restricted area of the animal's body was twisted. If the skeleton was completely hard (*e.g*. strongly mineralized), it could not be twisted. A simplified version of this scenario is presented (Fig. 9) in order to explain the observed deformation and twisting of some *Corumbella* specimens.

Although the theca of *Corumbella* was flexible, its tube-like body and its segments were not elastic, which is supported by the evidence that its structure was susceptible to breakage. For example, in the Tamengo Formation, *Corumbella* fossils are highly fragmented and fractured (Fig. 6C, D), including specimens that show evidence of syndepositional and/or pre-burial fragmentation and cracking. For instance, some fragments were broken prior to deposition, or are oriented inconsistently with the vertical compaction processes attributable to burial compaction. Similarly, abundant intricate specimens are sometimes concentrated in a single horizon, forming a pavement of *Corumbella* remains.

Considering these characteristics, thecae of *C*. *werneri* (Corumbá Group) may have been as flexible but not as functionally elastic as the periderm of modern coronates [39] and of some cnidarian fossils, like *Byronia* [40] or *Olivooides* [41] for example.

The coronate periderm is composed of thin lamellar layers of chitin [42] in a smooth microtexture arrangement [39, 43], functionally resulting in a soft tube that can be compressed and return to its original shape without damage or injury [39]. Morphologically, thecae of *C*. *werneri* also consist of a lamellar microfabric but made of polygonal plates (Figs. 6B, E and 7D, E); see ref. [17], like the Ordovician scyphozoan *Sphenothallus* that have integuments quite similar to some chitino-mineralized thecae of conulariids [17, 44, 45], and linguliids [46]. However, the theca of *Corumbella* is morphologically unique, because its polygonal plates sometimes have pores and papillae (Fig. 6F); see ref. [17].

The EDS mapping detected a higher concentration of calcium in remains of *Corumbella* thecae in comparison to the rock matrix (Fig. 7B). It suggests a biomineralized theca. But we have not ruled out the possibility that the calcium concentrations may be the result of diagenetic mineralization of organic tissue, rather than original biomineralization.

Nevertheless, skeletogenesis is the process of synthesizing skeletons and not necessarily involves biomineralization (which is the process that organisms precipitate solid materials to the formation of skeletal structures). Many animals synthesize completely organic skeletons, like insects, for example. Because our main issue is to investigate elements related to skeletogenesys process (including biomineralization as well), we also consider other elements related to cnidarian biomineralization, such as phosphate for example. However, we have not detected yet phosphate and other important elements related to this process besides calcium. We even had tried the chemical test for phosphate (1 dg of ammonium molybdate in reaction with 1:1 HNO3) [17] and it was not detected.

The ultrastructure of *Corumbella* also lacks the standard parabolic design characteristic of chitin-protein complexes, common to the former chitinous integuments exclusive from the Cambrian, such as the scyphozoan *Byronia robusta* [40, 47]. Moreover, unlike *Corumbella* and other current cnidarians, *Byronia* integuments are thinner, exclusively organic, and almost always preserved as carbonaceous films that are flattened but not fragmented [40, 47].

In summary, the plastic deformation of *C*. *werneri* suggests that its theca was flexible, organic, or even weakly mineralized. Furthermore, we hypothesize that the lamellar nature, thickness (and mineralization?) of its integument (Figs. 6B, E and 8E) may have contributed to its vulnerability to fragmentation (Fig. 6C, D). These characteristics suggest that the theca may have been originally thicker and less elastic than the periderms of modern coronates and *Byronia*, and that it was originally flexible but inelastic.

## Systematic Paleontology

The systematic review proposed here takes into account the approaches established for Brazilian specimens of *Corumbella werneri* [4–6, 12], which are applied to better constrain the systematic affinities of the incompletely described corumbellids from the lower member of the Wood Canyon Formation [9]. In order to facilitate this study and a comparison between Brazilian and USA specimens, we summarized data on the Brazilian specimens in a synoptic treatment, presented in Table 1.

**Table 1:** Revision of morphological terminology and taxonomic affinities of *Corumbella werneri*.

Description	Hahn *et al*. (1982)	Zaine (1991)	Babcock *et al*. (2005)	This study
**Body Organization**	Growth polarity	Growth polarity not evident	Growth polarity	Growth polarity
	Bipartite organization: composed of primary and secondary polyps	No bipartite organization	Description of oral region and observation of an apical attachment region	Observation of oral-aboral organization
			No secondary polyps	No secondary polyps
				Articulation and cross continuity of rings
**Geometry**	Uni- and biseriate cylindrical parts, circular in section	One to four longitudinal series of hollow flat, nearly cylindrical compartments	Elongate tube, square in cross-section	Elongated polyhedral tube (pyramidal), approximately quadrangular in cross-section
**Symmetry and internal thickening**	Sclerosepta only in primary polyp (tetraseptation)	Not observed	Carinae (attributed to the midline)	Presence of septa
				Carinae not observed
	Tetramery		Tetramery	Tetramery
**External structures**	No external structures (*e.g*. midlines and corners) corresponding to internal septations	Absent	Midline and corner	Midline, lateral edges and faces
**Covering**	Isolated rings in the proximal part	Isolated chitinous rings	Isolated rings	Rings are alternate across midline and continued at the lateral edges
	No rings in polypar			Thick carapace, sometimes with longitudinal striations
	Chitinous periderm		Possible chitinous periderm	Composition of carapace probably organic (chitinous?) or weakly mineralized
**Attachment structures**	Not observed	Not observed	Apical region attached to an organic mass	Apical region attached to the substrate
**Paleoecological considerations**	Colonial	Reproductive modes not evident	Reproduction by budding	Inferred gregarious and/or colonial habit
**Taxonomy**	Subclass Corumbellata, Order Corumbellida, Family Corumbellidae	Vendozoa, Vendobiont	Cnidaria, Scyphozoa, Conulata	Metazoa, Cnidaria, Scyphozoa,
**Phylogenetic relationships**	Cnidaria: *Stephanoscyphus*	Giant protists related to an extinct order (or subclass) of rhizopods	Cnidaria: Modern *Stephanoscyphus* and fossil conulariid	Cnidaria: fossil conulariid
	Pennatulacea, Charniidae			

Kingdom METAZOA Linnaeus 1758

Phylum CNIDARIA Verrill 1865

Class SCYPHOZOA Goette 1887

Family CORUMBELLIDAE Hahn, Hahn, Leonardos, Pflug e Walde 1982

Genus *Corumbella* Hahn, Hahn, Leonardos, Pflug and Walde 1982

Type species. *Corumbella werneri* Hahn, Hahn, Leonardos, Pflug and Walde 1982, by monotypy.

Emended diagnosis. Polyhedral, elongated tube, with internal septa formed by junction of alternate rings at midlines.

*Corumbella werneri* Hahn, Hahn, Leonardos, Pflug and Walde 1982

(Figs. 4–6 and 8)

*Corumbella werneri* Hahn et al., 1982: p. 4–9, Tables 1–3, Figs. 3–5, 9, 11.

*Corumbella* n. sp. Hagadorn and Waggoner 2000: Fig. 5 p. 356.

Emended diagnosis. Elongated polyhedral pyramidal exoskeleton (theca), thick, diameter along tube slightly variable; cross section circular at basal part, otherwise quadratic distalwards; external midline groove formed by junction of polygonal rings at apothem, continuous along the polyhedral tube; internal septa located internally to midline, when present; rings continuous over lateral edges, absence of carinae.

Description. Sample DGM-5601-I (Fig. 8C): three-dimensional internal mold, slightly compressed; tube "J"-shaped, 34 mm in height; aboral region of tube polyhedral, elongated, 9 mm in height; midline and lateral edges present. Sample GP/1E 4210a (Fig. 5E, F): internal mold of part of a three-dimensional polyhedral tube, quadrangular geometry, lateral edges visible; rings continuous at lateral edges; midline demarcated by merging of polygonal rings, in "U" conformation, with fragments of theca, alternately on face apothem (Fig. 5F); maximum length 9 mm, width 2 mm, maximum width of ring 1 mm, maximum length of ring 0.3mm. Sample DGM-5606-I (Fig. 8F): internal mold of part of a three-dimensional polyhedral compressed tube; lateral edges; rings continuous at lateral edges; midline demarcated by merging of rings alternately in face apothem, maximum length 19 mm, width 2.4 mm. Sample GP/1E 3093 (Fig. 8E) internal view of fragment compressed theca, thick; rings continuous at lateral edges; septa formed by merging of rings internally; thickness 0.05–0.2 mm, maximum length 9 mm. Sample GP/1E 4089 (Fig. 8A): oral region compressed at lateral edges, rings and lateral edges present; oral opening characterized by well marked hole. Sample GP/1E 4077 (Fig. 8B): tube inflated with three-dimensional rings, midline and septum present, lateral edges relatively compressed. Sample GP/1E 4204 (Fig. 8D): part of a three-dimensional polyhedral tube broken at the face and lateral edges; presence of polygonal rings; lateral edges and septa at midline visible in some parts of tube, absence of carinae. Sample LACMNH 12802 (Fig. 4A): three-dimensional polyhedral tube, presence of septa at midline and lateral edges; rings alternately converging at midline, apothem of face, twisting; specimen square in cross section; carinae absent.

Type series. DGM-5601I, DGM-5604I, DGM-5605I, DGM-5606I, DGM-5608I, DGM-5609I, DGM-5610I, DGM-5611I, DGM-5612I, DGM-5613I (Hahn et al., 1982, p. 4–9,Tables 1–3, Figs. 3–5, 9, 11), LACMNH 12802, LACMNH loc. 17130, LACMIP loc. 17130 (Hagadorn and Waggoner, 2000, p. 356, Fig. 5).

Examined Material. DNPM-RJ (DGM), 10 specimens; GSA/IGc-USP (GP/1E), 6 specimens; NHMLA (USA) (LACMNH, LACMIP), 2 specimens.

Discussion. All specimens studied here have similar morphology, and are summarized under the diagnosis of the genus *Corumbella*. The specimens from USA, which were previously assigned to “*Corumbella* n. sp.”, show longitudinal grooves at the midline and the alternated rings at the center of the polygonal faces. In the USA fossils, the midline is also indicated by the presence of its respective internal thickening (septum), which is visible in cross section. Septa are not always visible in all specimens, even in the Brazilian ones, a pattern that may be attributed to decay prior to burial. Comparisons between USA and Brazilian specimens showed that they can be considered the same species, *Corumbella werneri*, as evidenced by the presence of an external midline, formed by the alternation of polygonal rings at the apothem (Figs. 4A and 5F, H). This midline is continuous throughout the polyhedral tube, showing an unbroken continuity of the rings at the lateral edges (Figs. 4A and 5F-H). In considering these specimens, it is possible that the tube was occasionally expanded. Such expansion might have occurred since the flexible integument was susceptible to sediment infilling (maybe during life as in the case of some modern coronates) [50], or movement of the tube (Fig. 9). Hence, there is not a diagnostic and unique characteristic that justifies the assignment of a new species to the *Corumbella* from USA [9]; after comparisons based on [4]. Re-examination of these three-dimensional molds showed spaces between the molds and the rock matrix (Fig. 4A, G), which indicates the previous existence of a relatively thick theca—one that would be similar in thickness to the skeletal integument of the specimens from the Tamengo Formation of Brazil and the Tagatiya Guazu Formation of Paraguay. The quadrangular cross-section along the length of the tube (Figs. 4A, F and 5D, H) reinforces our interpretation that the skeletal structure of *Corumbella* was more resistant than many fossils that are always found flattened or compressed but never fragments or with breaks (such as *Byronia*).

## Conclusions and Future Directions

Our data not only confirms previously published observations [5, 9], but also add new information about morphology (*e.g*. attachment portion, oral region, rings disposition and midline) and ultrastructure (*e.g*. lamellae microfabric) that contributes an important complement to the interpretation and a new description of *C*. *werneri*.

This study is consistent with the idea that *Corumbella* is a scyphozoan cnidarian and that specimens from Brazil, Paraguay, and the USA are part of a similar taxonomic and benthic assemblage. The occurrence of specimens with the tip of *Corumbella*’s tube-like skeleton embedded in the sediment suggests that their mode of life was possibly similar to that of helicoplacoids, in which the animal was attached to or rooted in the substrate. *Corumbella* also had a relatively thick and sturdy theca that showed complex growth and flexure.

The taphonomic aspects considered here are only those essential to better perform morphological studies (*e.g*. distortion, compression) refusing bias in taxonomic descriptions. As the flexible exoskeleton of *Corumbella* may be susceptible to molding processes and/or sediments filling, we performed some insights into the importance of some deformations (*e.g*. twisting) to the interpretation of how some taphonomic classes, such as three-dimensional, can be preserved and incur in important specimens for taxonomic descriptions. Here we only offer a model that justifies the twists observed in *Corumbella* are not intrinsic to the organism (morphological) but an artifact of deformation related to external forces on its polyhedral structure during burial (see Fig. 9).

In this sense, some causes of the preservational style of its theca still remain enigmatic. The distribution of *Corumbella* remains among the marls and shales of Tamengo Formation lacks satisfactory paleoenvironmental explanation and taphonomic models. Detailed taphonomic studies (involving the compilation of a deep sedimentological database) are still crucial to reveal fundamental aspects of fossilization process in *Corumbella* specimens.

In addition, further research about the possible mineralized nature (phosphatic or calcitic) of specimens may shed light on the corumbellids’s role in the development of metazoan biomineralization.
